# Supplementary material for: HOP1 and HAP2 are conserved components of the meiosis-related machinery required for successful mating in Leishmania
Source: Nat Commun. 2023 Nov 7;14:7159. doi: 10.1038/s41467-023-42789-z (PMC10630298; doi:10.1038/s41467-023-42789-z)
Supplement: Supplementary file 1 — Supplementary Information [file 41467_2023_42789_MOESM1_ESM.pdf]

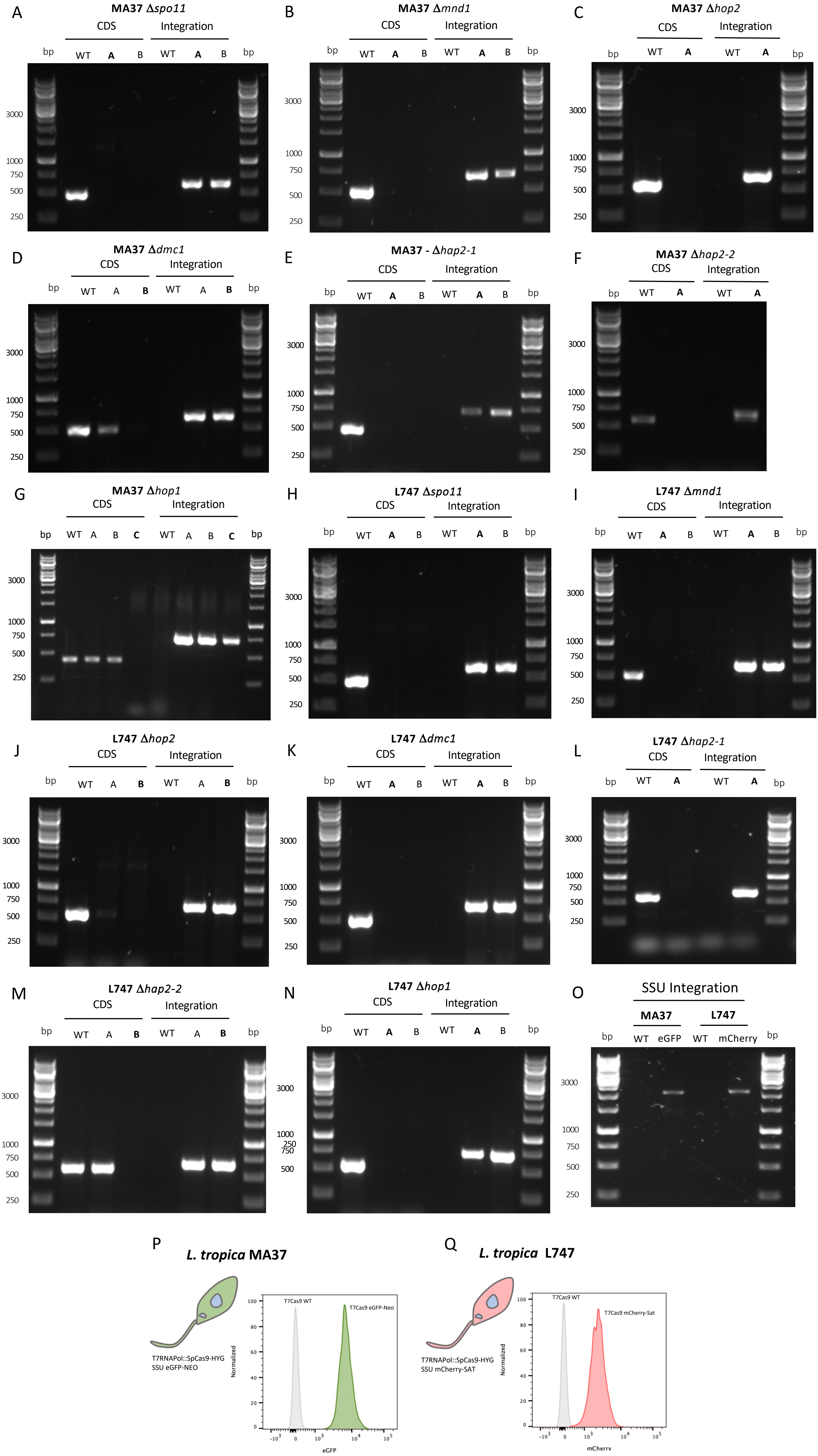

Supplementary Fig. 1. *Leishmania tropica* cell lines confirmation. The non-fluorescent MA37 T7Cas9 and L747 T7Cas9 were the background cell lines used to generate the null mutants for meiosis-related genes. (A-N) PCR confirmation that all genes of interest could be deleted in promastigotes *in vitro*. Strains and deleted genes are depicted in the figure above each agarose gel. Letters on top of each lane represent a clone tested by diagnostic PCR. All oligonucleotides used for diagnostic PCR are in Supplementary Table 4. (O) Representative agarose gel for integration of the fluorescent proteins in the small subunit of the ribosome (SSU) of control cell lines MA37 and L747 T7Cas9. (P) MA37 T7Cas9 eGFP-Neo and (Q) L747 T7Cas9 mCherry-Sat cell lines expressed the desired fluorescent proteins as confirmed by cytometry.

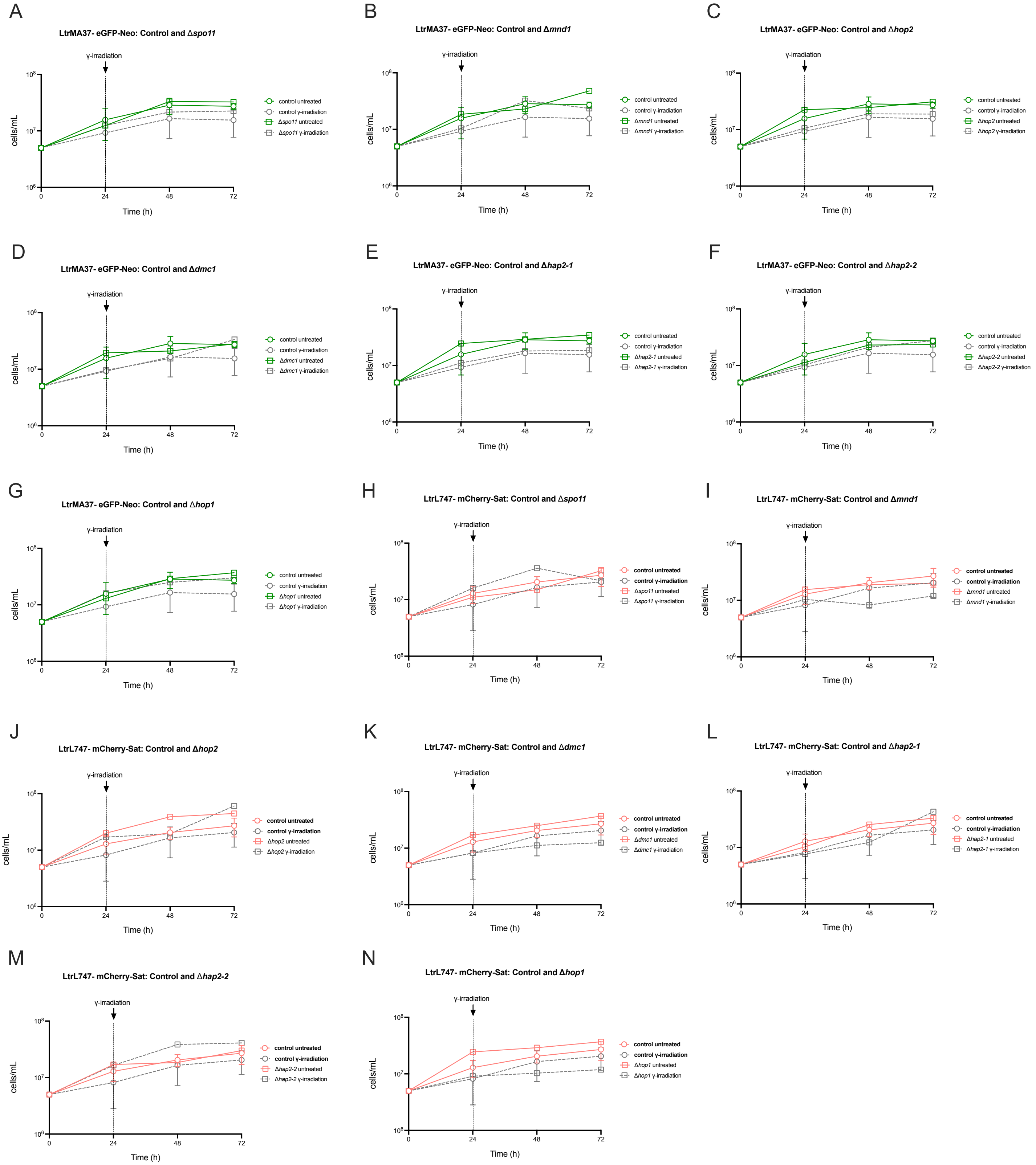

Supplementary Fig. 2. *Leishmania tropica* promastigote proliferation is not affected by the deletion of meiosis-related genes or by γ-irradiation. Panels A-G represent strain MA37, showing proliferation profiles of untreated (green lines) and γ-irradiated (grey lines) cultures for control (T7Cas9) and null mutants of meiosis-related genes (A) *SPO11*; (B) *MND1*; (C) *HOP2*; (D) *DMC1*; (E) *HAP2-1*; (F) *HAP2-2*; (G) *HOP1*. Panels H-N represent strain L747, showing proliferation profiles of untreated (red lines) and γ-irradiated (grey lines) cultures of control (T7Cas9) and null mutants of meiosis-related genes (A) *SPO11*; (B) *MND1*; (C) *HOP2*; (D) *DMC1*; (E) *HAP2-1*; (F) *HAP2-2*; (G) *HOP1*. The dotted line and arrow on each graph indicate the time cultures were irradiated. Mutants were individually assessed by cell counting every 24 hours. Mean ± SEM of 2 independent experiments. *p* values (non-significant) were calculated by one-way ANOVA with multiple comparisons of pre-selected pairs corrected with Šidák test.

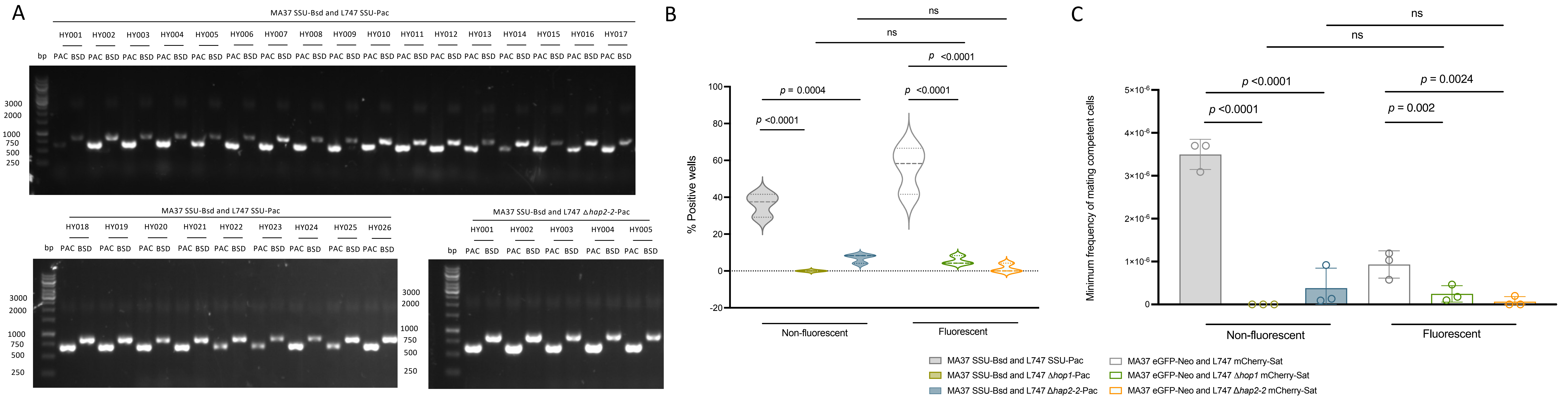

Supplementary Fig. 3. Comparison of *HOP1* and *HAP2-2* null mutants hybridization defect of non-fluorescent and fluorescent cell lines. (A) Agarose gel for confirmation of integration of both antibiotic resistance genes in hybrids recovered from *in vitro* crosses of MA37 SSU-Bsd and L747 SSU-Pac (controls) and MA37 SSU-Bsd and L747 Δ*hap2-2*-Pac. (B) Violin plots of the percentage of positive wells comparing crosses of non-fluorescent (Bsd and Pac) and fluorescent (Sat and Neo) lines. (C) Bar graphs representing the minimum frequency of hybridization competent cells comparing crosses of the non-fluorescent and fluorescent cell lines shown in (B). Results are represented as the mean of 3 independent experiments ± SD. *p*-values determined by one-way ANOVA for multiple comparison of preselected pairs and a two-step step-up method of Benjamini, Krieger and Yekutieli to correct false-discovery.

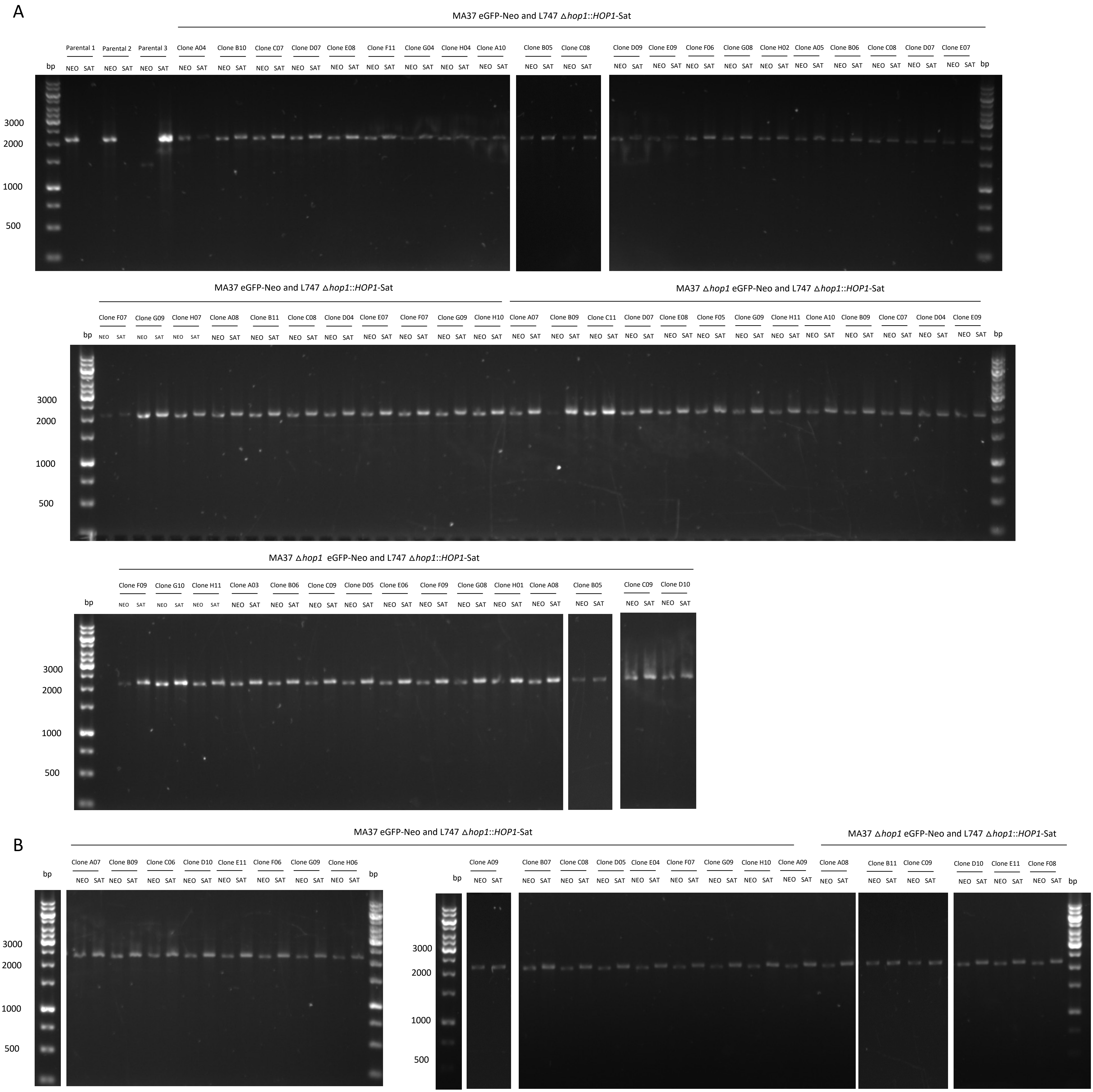

Supplementary Fig. 4. Confirmation of correct integration of resistance markers Neo and Sat in parental cell lines and hybrid clones generated *in vitro* (A) and *in vivo* (B). Each clone represents an independent hybrid recovered from a single well. Parental cell line 1: MA37 eGFP-Neo; Parental cell line 2: MA37 Δ*hop1* eGFP-Neo; Parental cell line 3: L747 Δ*hop1*::*HOP1*-Sat.

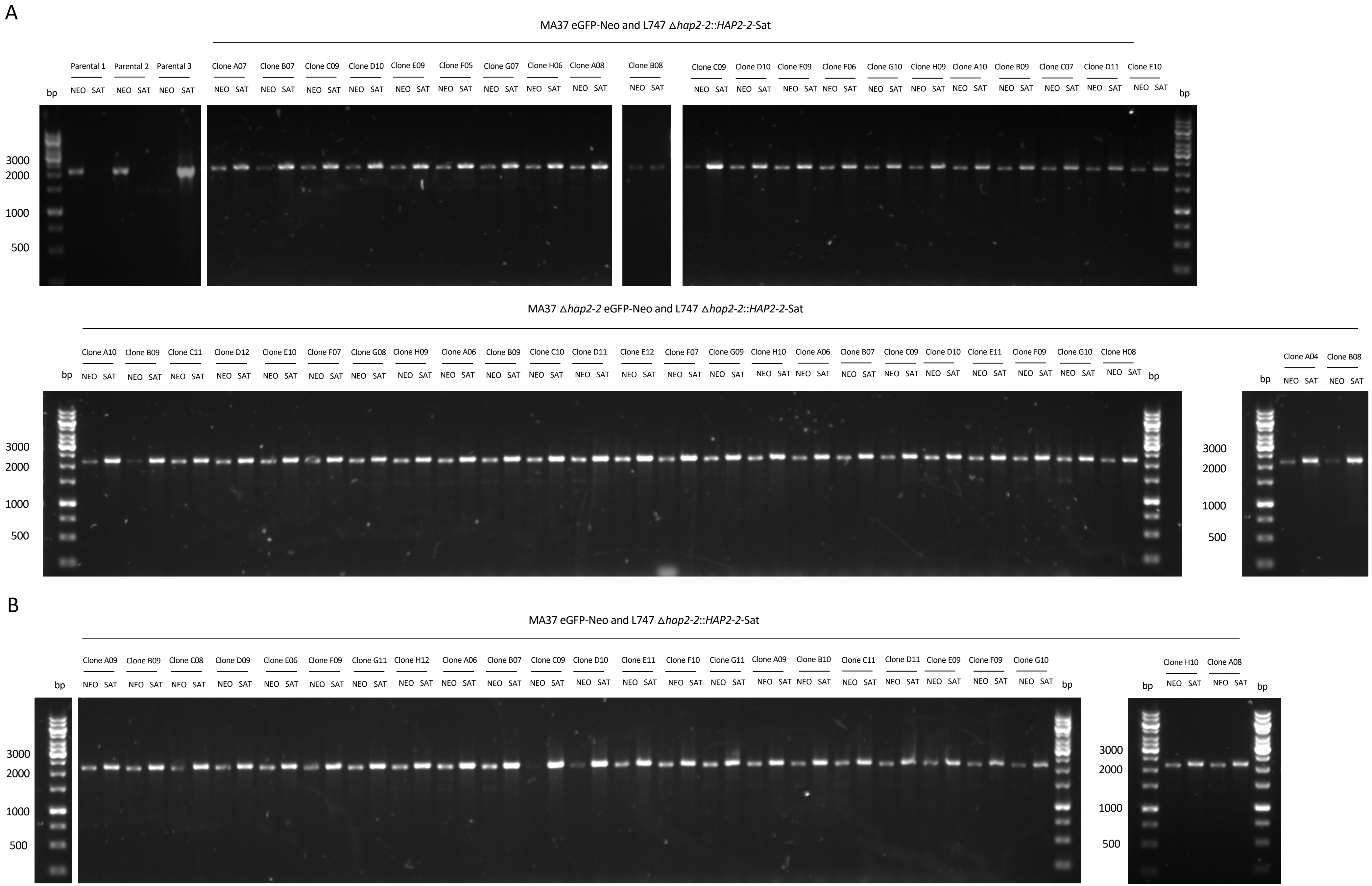

Supplementary Fig. 5. Confirmation of correct integration of resistance markers Neo and Sat in parental cell lines and hybrid clones generated *in vitro* (A) and *in vivo* (B). Each clone represents an independent hybrid recovered from a single well. Parental cell line 1: MA37 eGFP-Neo; Parental cell line 2: MA37 Δ*hap2-2* eGFP-Neo; Parental cell line 3: L747 Δ*hap2-2*::*HAP2-2*-Sat.

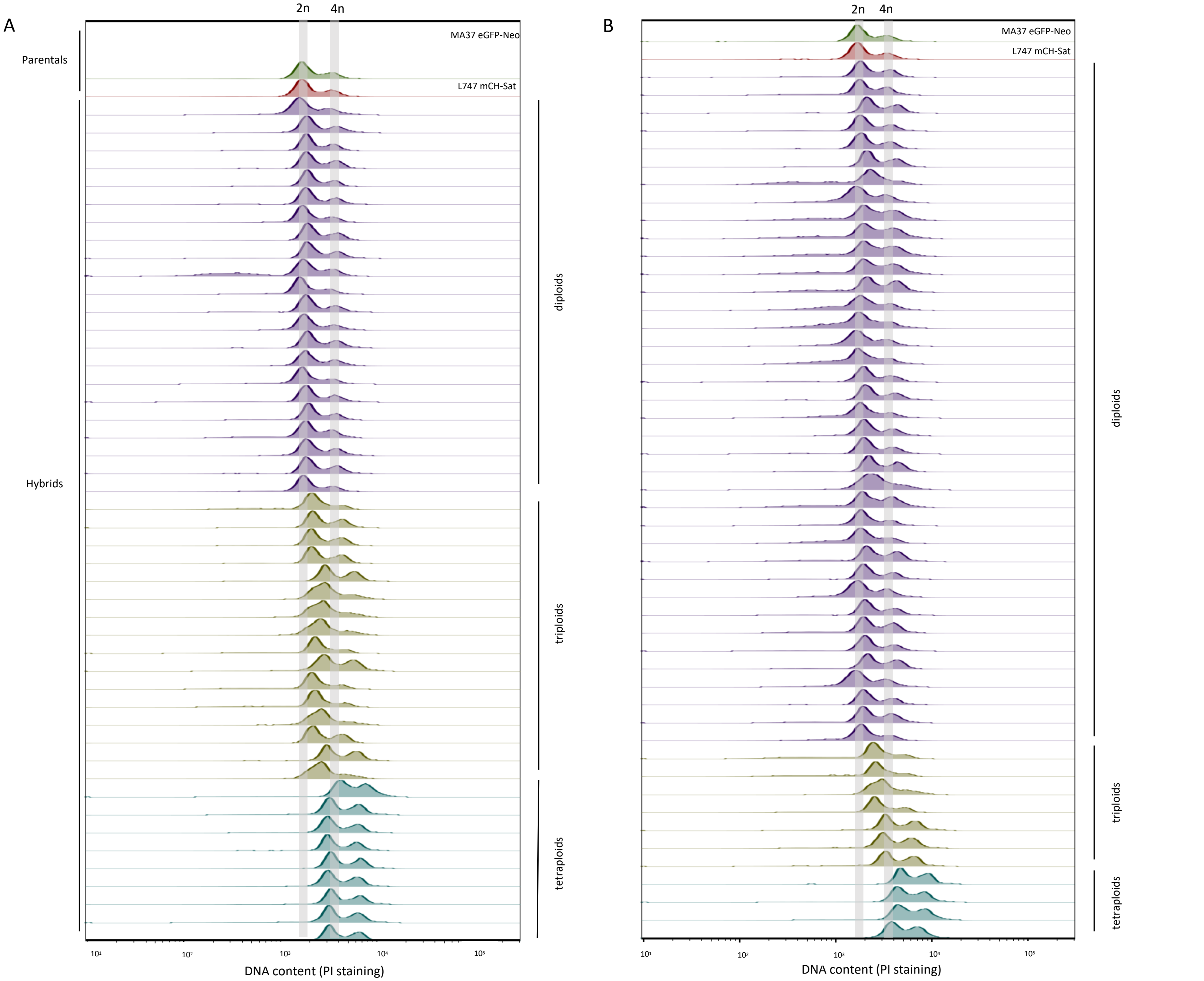

Supplementary Fig. 6. DNA content analysis was performed using propidium iodide (PI) staining and flow cytometry on both parental strains and control hybrids (MA37 eGFP-Neo and L747 mCherry-Sat). In (A), controls for experiments presented in Figure 4 (HOP1 hybrids) are displayed, and in (B), controls for experiments in Figure 5 (HAP2-2 hybrids) are shown. The grey bands indicate the DNA content corresponding to 2n (G1/G0 peak) and 4n (G2/M peak) in parental strains.

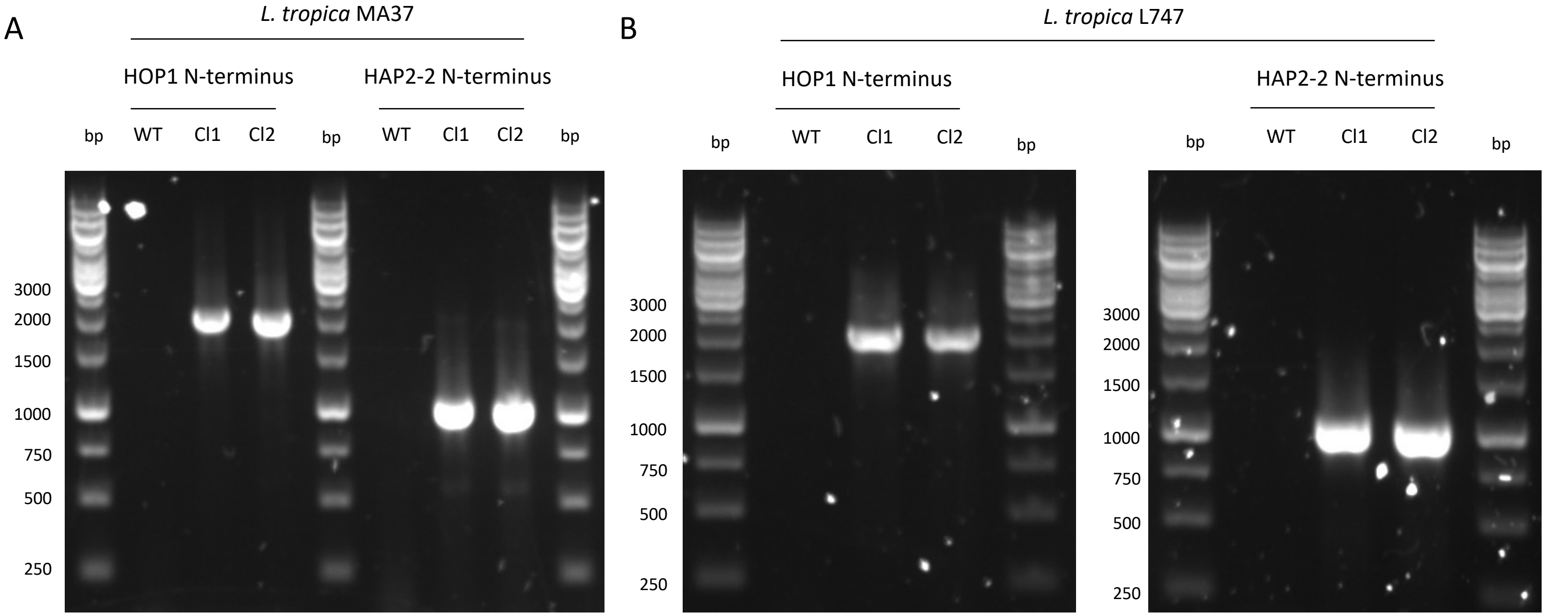

Supplementary Fig. 7. Confirmation of repair cassette containing mNeonGreen and Bsd resistance gene at the 5' end of *HOP1* and *HAP2-2* in (A) MA37 and (B) L747 reporter lines.
