# Supplementary material for: HOP1 and HAP2 are conserved components of the meiosis-related machinery required for successful mating in Leishmania
Source: Nat Commun. 2023 Nov 7;14:7159. doi: 10.1038/s41467-023-42789-z (PMC10630298; doi:10.1038/s41467-023-42789-z)
Supplement: Supplementary file 2 — Description of Additional Supplementary Files [file 41467_2023_42789_MOESM2_ESM.pdf]

Description of Additional Supplementary Files

File Name: Supplementary Data 1
Description: Calculation of minimum frequencies of hybridization and percentage of positive wells for in vitro crossing experiments.

File Name: Supplementary Data 2
Description: Raw data for sand fly crossing experiments, including total number of infected midguts, % of hybrids recovered, and ploidy quantification (PI staining) of hybrids.

File Name: Supplementary Data 3
Description: Heterozygous and homozygous SNP counts (raw data from Fig. 4E and 5D), sequencing quality and coverage of WGS samples, and mean parental chromosomal contribution in parental lines and hybrids.

File Name: Supplementary Data 4
Description: Oligonucleotides, cell lines and constructs used in this study.

File Name: Supplementary Movie 1
Description: *L. tropica* L747 mNG::HOP1 infection of *Lu. longipalpis* midgut. Video showing no expression of HOP1 by promastigotes in the promastigote secretory gel plug.

File Name: Supplementary Movie 2
Description: *L. tropica* L747 mNG::HAP2-2 (green) infection of *Lu. longipalpis* abdominal midgut. Elongated cells with long flagella resembling metacyclic promastigotes expressing HAP2-2 diffusely in the cytoplasm and in an intracellular organelle. Smaller rounded cells expressing HAP2-2 on the surface.
